# Modelling the Meteorological Forest Fire Niche in Heterogeneous Pyrologic Conditions

**DOI:** 10.1371/journal.pone.0116875

**Published:** 2015-02-13

**Authors:** Antonella De Angelis, Carlo Ricotta, Marco Conedera, Gianni Boris Pezzatti

**Affiliations:** 1 WSL Swiss Federal Research Institute, Insubric Ecosystems Research Group, via Belsoggiorno 22, CH-6500, Bellinzona, Switzerland; 2 Department of Environmental Biology, University of Rome “La Sapienza”, Piazzale Aldo Moro 5, 00185, Rome, Italy; Oregon State University, UNITED STATES

## Abstract

Fire regimes are strongly related to weather conditions that directly and indirectly influence fire ignition and propagation. Identifying the most important meteorological fire drivers is thus fundamental for daily fire risk forecasting. In this context, several fire weather indices have been developed focussing mainly on fire-related local weather conditions and fuel characteristics. The specificity of the conditions for which fire danger indices are developed makes its direct transfer and applicability problematic in different areas or with other fuel types. In this paper we used the low-to-intermediate fire-prone region of Canton Ticino as a case study to develop a new daily fire danger index by implementing a niche modelling approach (Maxent). In order to identify the most suitable weather conditions for fires, different combinations of input variables were tested (meteorological variables, existing fire danger indices or a combination of both). Our findings demonstrate that such combinations of input variables increase the predictive power of the resulting index and surprisingly even using meteorological variables only allows similar or better performances than using the complex Canadian Fire Weather Index (FWI). Furthermore, the niche modelling approach based on Maxent resulted in slightly improved model performance and in a reduced number of selected variables with respect to the classical logistic approach. Factors influencing final model robustness were the number of fire events considered and the specificity of the meteorological conditions leading to fire ignition.

## Introduction

A fire regime is the result of complex interactions between weather conditions, fuel, topography and ignition sources [1], [2], [3]. Climatic factors have a long term effect on the type and amount of biomass production, related fuel availability and a short term influence on actual vegetation moisture and related flammability, as well as fire propagation conditions (e.g. wind) [4], [5]. In case of lightning, weather conditions may also act as direct ignition source [6]. Similarly, humans may indirectly influence fire regimes through land use by shaping the type, amount and connectivity of vegetation and related fuel [7], through fire management activities such as fire legislation, fuel regulation and fire fighting activities, as well as by directly acting as intentional or unintentional ignition sources [8], [9], [10]. As a result, fire regimes always have natural and anthropogenic components, although with varying relative contributions [1], [7].

Understanding the main fire drivers and their variations within a specific region is the main objective of modern fire and environmental management agencies that are in charge of the appropriate allocation of fire fighting resources from day to day or place to place [11], [12]. The process of systematically evaluating individual or combined factors influencing fire potential is usually referred to as fire danger rating [13]. The aim of fire danger rating systems consists of integrating scientific knowledge about the reaction of specific forest fuels to weather conditions into models that are based on a representative set of consistently measurable fire-related variables [13]. Consequently, a number of approaches have been designed around the world, all sharing the same basic idea of synthesizing environmental and meteorological conditions that affect ignition, spread, and effort to control fires into qualitative and/or numerical fire danger indices [14], [12]. Such fire danger indices provide a measurement of the chance that a fire will start from a particular fuel, its rate of spread, intensity and difficulty to suppress, through various combinations of temperature, relative humidity, wind speed and drought effects.

As originally stated by van Wagner [15], a fire danger index should ideally work so that any given index value will always represent the same fire behavior, no matter what weather history leads up to it. Fuel wets and dries and fire responds to variations of fuel, weather and topography according to the same physical processes and principles regardless of location [13]. This theoretically gives to indices that are based on simple and universal moisture exchange models a great adaptive potential to different fire environments worldwide [11]. Thus, for instance, the Canadian FWI and related sub-indices that are based on sound scientific principles have outstanding interpretative support, and have therefore been implemented and adapted in many countries or regions all around the world [16], [17], [13], [18], [19], [20], [21].

The specificity of the conditions for which fire danger indices are developed, however, makes their practical adoption problematic in areas or with fuel types other than those for which they were intended [22], [11]. On the other hand, the adoption of existing fire danger rating systems can significantly reduce the time and the costs involved in developing a rating approach in a new area of application. Important calibration steps may be necessary in order to cover the full fluctuation range of daily fire conditions, as well as to assure a sound correlation between fire danger index outputs and the historical fire activity in the area of new application [16]. This is particularly important—but also accordingly difficult—in areas where a fire regime has a strong anthropogenic component in terms of fuel regulation, ignition source or fire control activities. In the case of fire bans or of evident and elevated fire danger, unintended human-induced fire ignition may not follow, causing a false non-fire day that may influence the calibration of physically-based fire danger indices. The fire community has attempted to overcome this problem by implementing extended modelling approaches [23], [24], [25] and by recalibrating index output scales [26], [18], [21], [27], [28]. So far, however, no fully satisfactory and universally applicable calibration method has been proposed.

In this paper we propose a new approach for developing a daily fire danger index at the regional scale consisting of:

- considering combinations of meteorological parameters, fire weather indices or both as input variables for modelling;
- applying the principle of ecological niche modelling to a temporal scale in order to identify specific daily fire weather conditions.

We hypothesize, on one hand, that building a model that takes different fire weather indices and meteorological variables into consideration may make it possible to better consider the specific fire relevant weather conditions of the study area with respect to a model based on single fire weather indices, and, on another hand, that using presence data (days with fire occurrence) only in the frame of a niche modelling approach may enhance the identification of fire-prone climatic variables and the performance of related fire danger ratings. This may be especially the case in a highly anthropogenic and heterogeneous fire environment where overtly obvious fire conditions may result in no ignition by humans. To test these hypotheses, we consider the time line (each day of a fire regime) as a spatial environment where existing fire weather parameters act as environmental parameters (explanatory variables) that shape the suitable “fire niche”. Historical fire occurrence (fire days) acts, in turn, as a response variable. Among existing niche modelling approaches, we selected the machine learning-based Maxent [29] because of its low sensitivity to the number of presences [30], [31]. The efficiency of the Maxent approach was then tested by comparing its performance with a generalized linear model (GLM) with binomial distribution and logistic link, as traditionally used for forest fire predictions [32], [33], [34].

Both modelling approaches were tested using a combination of meteorological variables, fire weather indices, and the combination of both as explanatory variables. The resulting best models were then compared with the worldwide most adopted single fire weather indices.

We chose the low-to-intermediate fire-prone region of Canton Ticino in southern Switzerland as a study case because of the presence of anthropogenic and naturally driven seasonal fire regimes as well as the very heterogeneous fuel conditions in the region.

## Materials and Methods

### Study area

**Fig 1:**
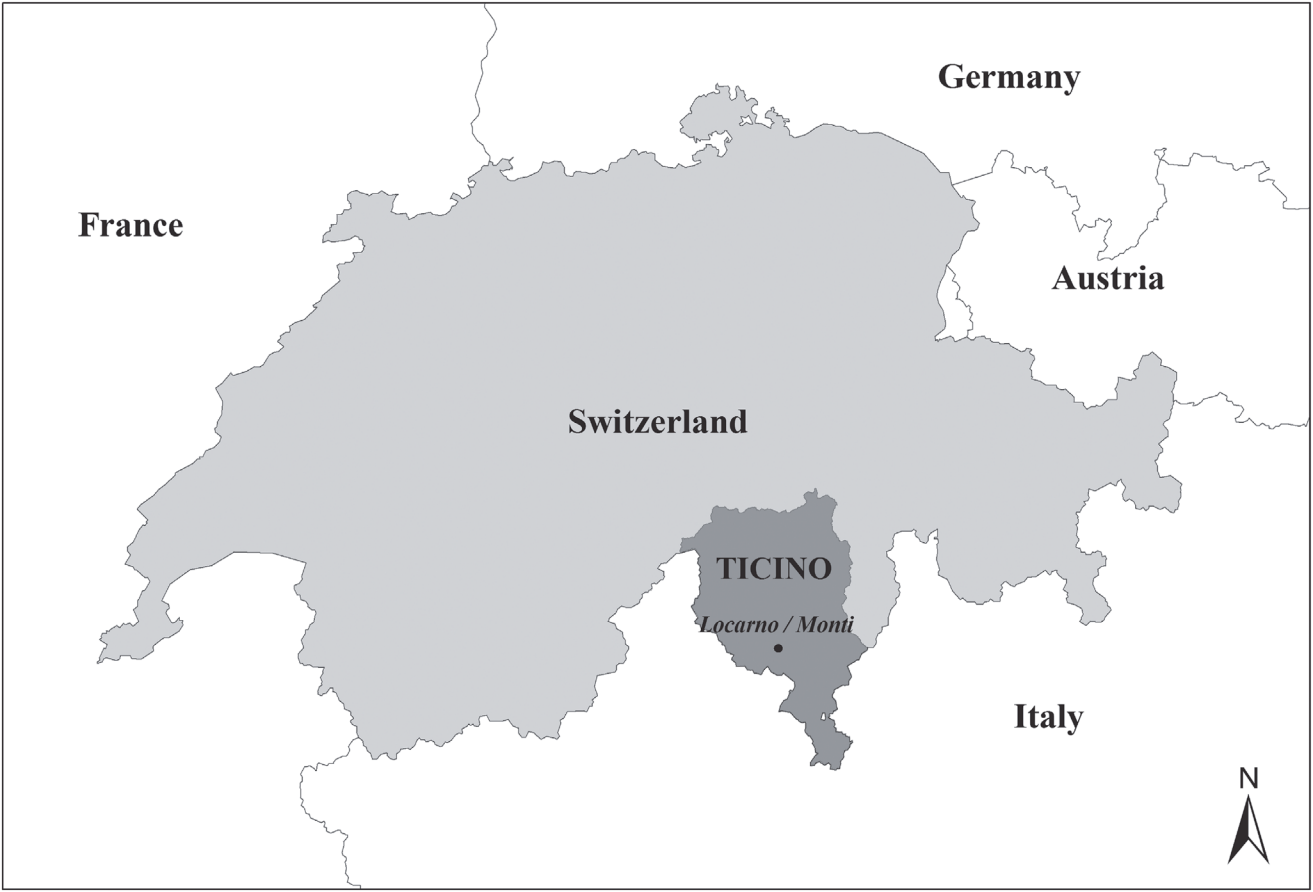
The study area of Canton Ticino (Switzerland) with the meteorological station of Locarno/Monti.

The study area is represented by the Canton Ticino (Switzerland) (Fig. 1) a mountainous Swiss canton that ranges from 200 m to 3,400 m a.s.l. Summers are wet and warm and winters dry and mild (‘Insubrian’ climate). The mean annual precipitation ranges from 1,660 to 2,600 mm and is mostly concentrated between June and September while the mean annual temperature ranges from 3 to 12°C (source: MeteoSwiss climatic norm values 1981–2010).

The climate is often influenced by a dry foehn wind from the north, which occurs occasionally in the main valleys of the study area causing significant drops in the relative air humidity (down to 20%) [35].

About 50% of the total surface of Canton Ticino is covered by heterogeneous structured forest with the species composition that reflects the altitudinal gradient. The early introduced Sweet chestnuts (*Castanea sativa*) at low elevations [36], with an occasional mixture by other broadleaved species such as, deciduous oak (*Quercus* spp.), small-leaved lime (*Tilia cordata*), wild cherry (*Prunus avium*), black alder (*Alnus glutinosa*), maple (*Acer* spp.), and ash (*Fraxinus* spp.). The slopes at medium elevations (900–1,400 m a.s.l.) are dominated by European beech (*Fagus sylvatica*) forests, followed by Norway spruce (*Picea abies*) stands [37] intermixed with European silver fir (*Abies alba*), pine species (*Pinus spp*.), and European larch (*Larix decidua*).

### Forest fire data and current fire regimes

Fire data has been collected in Ticino by the Forest Service since 1900, and geo-referenced perimeters of the burnt area exist for most forest fires since 1969 [38]. According to Pezzatti et al. [10], a significant shift in burnt area took place—starting in the 1980s—as a result of a major fire brigade reorganisation (1978) and of the start of the systematic use of helicopters for both the transport of fire fighters and aerial fire fighting. Concerning fire frequency, a relevant drop in anthropogenic induced fire ignitions without a corresponding change in the precipitation regime occurred in 1990 as a consequence of two preventative legal acts [36], [10]: the prohibition of burning garden debris in the open (Cantonal decree approved on October 21, 1987, but operational with the corresponding penalties since January 1, 1989) and the prohibition against fireworks and celebration fires on the Swiss National Day of August 1st in case of high fire ignition danger (Cantonal decrees of July 11, 1990). Present pyrologic conditions have existed therefore since 1980 concerning burnt areas and since 1990 concerning the anthropogenic fire ignitions [10].

**Fig 2:**
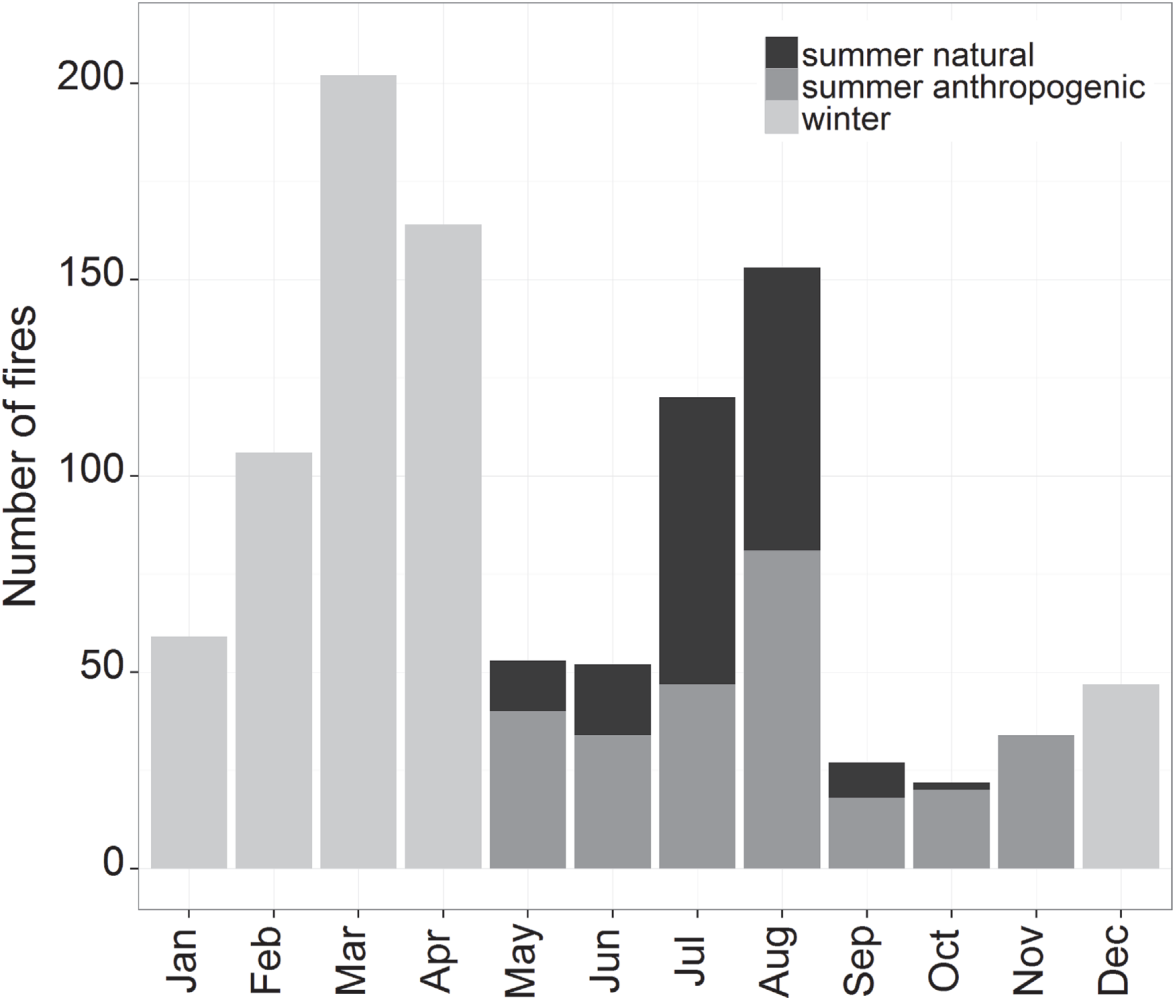
Monthly fire distribution of Canton Ticino. Monthly values refer to the same database used for the analysis: the winter fires from 1991 to 2012 (light grey), the summer anthropogenic fires from 1991 to 2012 (grey) and the summer natural fires from 1981 to 2012 (dark grey).

The current monthly distribution of fires highlights the existence of three fire regime patterns (Fig. 2). During vegetation rest (December to April, hereafter called winter) fire events mostly consist of rapid spreading (surface) fires of anthropogenic origin with a major peak in March-April. During the vegetation season (May-November hereafter called summer) and in the summer months of July-August in particular, a mixed pattern consisting of slow spreading fires of both natural (lightning-induced) and anthropogenic origin (hereafter called summer natural and summer anthropogenic respectively) dominates. According to Conedera et al. [39], summer fires also have two distinct geographic distributions: lightning-induced fires are concentrated in the coniferous forests at higher altitudes and on steeper slopes than human-caused events.

In the present study, homogeneous fire regime conditions in the study area refer to the period 1991–2012 for both winter (w) and summer anthropogenic fires (sa) and 1981–2012 for natural summer fires (sn) (Fig. 2). Extending the period of the natural forest fire regime back to 1981 represents a compromise between the consistency (homogeneity) and the representativeness (quantity) of the data, since lightning-induced fire ignitions are not influenced by preventive legislation.

### Meteorological data and fire weather indices

Daily meteorological data was gathered from the MeteoSwiss meteorological station of Locarno-Monti, which is considered to be representative for the whole study area (Fig. 1).

The daily meteorological variables used are: Air Temperature in Celsius degree (T), Air Humidity in percentage value (H), Wind velocity in m/s (U), Precipitation in mm (P), coverage of sky in ratio between 0 and 1 (CloudCover) and coverage of snow expressed as snow presence or absence (SnowCover). From the original data, we also derived some cumulative meteorological parameters to be used as model input variables: total rainfall over the last seven days (WeekRain), the days since the last rainfall (DaysSinceRain), the sum of the last rainfall (consecutive days with rain) (LastRainSum), the dew point temperature (Tdew), that is the temperature to which air needs to be cooled to make air water vapour saturated [40] and the vapour pressure deficit (VPD) that is the difference between the saturation vapour pressure and the actual vapour pressure at a particular temperature.

The meteorological variables were used as input for the *Fire Weather Indices Calculator* software, developed in the frame of the ALPFFIRS project by WSL (www.wsl.ch), in order to calculate fire weather indices for each day of the time interval considered (Table 1). In this study, we retained 15 of the most well-known fire indices used to assess forest fire hazards in several parts of the world (see Table 1 for more details).

**Table 1:** Fire indices used in this study and related input variables, meteorological data and site parameters used for calculations.

Fire indices	Acronym	Daily meteorological data							Other meteorological data		Site parameters		single indices for comparison (*)	References
		T	Tmax	Tmin	Tdew	H	P	U	Mean monthly T	Mean annual sum of P	Elevation	Latitude		
Angström index	Angstroem	[°C]				[%]							x	[67]
Baumgartner index	Baumgartner	[°C]	[°C]	[°C]			[mm]	[m/s]			[m a.s.l.]	[rad]		[68]
Fine fuel moisture code	FFMC	[°C]				[%]	[mm]	[km/h]					x	[15]
Duff moisture code	DMC	[°C]				[%]	[mm]						x	[15]
Drought code	DC	[°C]					[mm]						x	[15]
Initial spread index	ISI	[°C]				[%]	[mm]	[km/h]						[15]
Buildup index	BUI	[°C]				[%]	[mm]							[15]
Fire weather index	FWI	[°C]				[%]	[mm]	[km/h]					x	[15]
Fosberg fire weather index	FFWI	[°C]				[%]		[mph]						[69]
Keetch-Byram drought index	KBDIsi	[°C]					[mm]			[mm]			x	[70]
Mc Arthur Mark 5 forest fire danger index	FFDI	[°C]				[%]	[mm]	[km/h]		[mm]				[71]
Munger drought index	Munger						[in]							[72]
Orieux index (danger scale)	Orieuxdanger	[°C]					[mm]	[km/h]	[°C]			[rad]		[73]
Nesterov ignition index	Nesterov	[°C]			[°C]		[mm]						x	[74]
Sharples fuel moisture index	FMI	[°C]				[%]							x	[75]

When a variable is retained, the units are in square brackets.

(*) single indices used as input for logistic models for the comparison with the best Maxent models (see Fig. 4 and Table 4).

In order to consider all existing fire regimes, meteorological and fire data were split according to the three fire regimes: winter (w), summer anthropogenic (sa) and summer natural (sn). Within the three fire regimes we defined the days with at least a fire ignition of the correspondent origin (anthropogenic or natural) as “fire days”, and the ratio between fire days and the total number of the days considered as “prevalence” (hereafter called background, see Table 2).

**Table 2:** The total number of fire days (days with at least one fire ignition corresponding to presence in the niche models) and background (the total number of the days) in the time interval considered and the prevalence value calculated as the ratio between the two previous numbers for each fire regime.

Time interval	Regime	Fire days	Background	Prevalence
1991–2012	w	444	3328	0.133
	sa	163	4708	0.035
1981–2012	sn	151	6848	0.022

Three different sets of input variables were then defined: the meteorological variables (hereafter called meteo), the fire weather indices (hereafter called indices), and the combination of a subset of both (hereafter called mixed).We decided to test also the combination of climatic variables and indices, in order to take into account both the linear information of the single meteorological variables and the complex, non-linear or cumulative information that the same variable may reflect within the complex algorithm of a fire index. To avoid redundancy, however, we retained only the combinations with all pairwise Spearman correlations among the variables lower than 0.9. See *input variables* in Table 3 for more details.

**Table 3:** Selected variables and model performances for the input variable combinations and fire regimes considered.

REGIME	Maxent BEST MODELS	Input meteorological variables										
		T [°C]	Tdew [°C]	P [mm]	U [m/s]	H [%]	VPD [kPa]	CloudCover [ratio 0/1]	Weekrain [mm/week]	LastRainSum [mm]	DaysSinceRain [day]	SnowCover [0/1]
**w**	**meteo**	○	●	●	○	●	○	●	●	●	○	●
	**indices**											
	**mixed**	○	○	●	○	○		●	○			●
**sa**	**meteo**	●	○	○	●	○	●	○	●	○	●	
	**indices**											
	**mixed**	○	○	○	○	○		○	○			
**sn**	**meteo**	○	○	○	●	●	●	●	●	○	●	
	**indices**											
	**mixed**	○	●	○	●	●		○	○			

REGIME	Maxent BEST MODELS	Input indices															Performances	Prevalence-based theoretical Maximum AUC.bg
		Angstroem	Baumgartner	FFMC	DMC	DC	ISI	BUI	FWI	FFWI	KBDIsi	FFDI	Munger	Orieuxdanger	Nesterov	FMI	Mean k-folds and Range of the AUC.bg	
**w**	**meteo**																0.7655 (0.6941–0.8519)	0.9333
	**indices**	○	○	○	●	○	○	○	○	○	○	○	○	●	●	●	0.7776 (0.714–0.869)	0.9333
	**mixed**		○		●				○	○		○	○	●	●	●	0.7789 (0.7228–0.8765)	0.9333
**sa**	**meteo**																0.7516 (0.5829–0.8405)	0.9827
	**indices**	○	○	○	●	○	○	○	○	●	○	●	●	●	○	○	0.7716 (0.6261–0.8734)	0.9827
	**mixed**		○		●				○	●		●	●	●	○	○	0.7716 (0.6261–0.8734)	0.9827
**sn**	**meteo**																0.832 (0.6577–0.9375)	0.9890
	**indices**	○	●	○	○	○	○	○	●	○	○	○	●	●	○	●	0.839 (0.6231–0.9585)	0.9890
	**mixed**		●		○				●	○		○	●	●	○	○	0.8517 (0.6259–0.976)	0.9890

Considered unselected (○) and selected variables (●) for the best Maxent models are shown.

### Models

As term of reference we used the logistic model. It is a particular case of the generalized linear model (GLM—[41]), also widely used in species distribution modelling because of its strong statistical foundation and ability to realistically describe ecological relationships [42], [30]. Generalized linear models are extensions of linear regression models that can handle non—normal distributions such as binomial distributions (e.g. presence-absence data; [43], [44]). GLMs are based on an assumed relationship, called a link function, between the mean of the response variable and the linear combination of the explanatory variables [43]. In our case, the link function is the “logit” function of the binary response data, and therefore the appropriate GLM is a logistic model. Each day of the study period and of the corresponding fire regime represents a binary response variable for which we considered the days with at least one fire ignition (fire days) as *presence* and the days without fire ignitions as *absence*.

Among existing niche modelling approaches, Maxent is a machine learning method that uses the principle of maximum entropy on presence data to estimate a set of functions that relate environmental variables and habitat suitability, so that it approximates the species’ niche and potential geographical distribution [29]. The principle of maximum entropy is used to seek a marginal suitability function for each variable that matches the empirical data and is maximally uninformative elsewhere [45]. The functions of the environmental variables are called features [29] and may be linear, product, quadratic, hinge, threshold or categorical. The use of these functions makes it possible to consider the complexity and potential non-linearity of the species’ response to environmental factors [42]. An increasing number of features building the model is treated as a penalty to avoid creating complex and over-fitted models. In order to avoid over-fitting [45], Maxent software uses a regularization process that allows model distributions to lie in a range around empirical data.

Widely used for many purposes such as biogeography and ecology, Maxent has recently been applied to wildfires to model fire occurrence in India’s Ghats Mountains [46] and fire ignition and distribution in the US [47], [34]. In the present study, we test the efficiency of Maxent in considering daily meteorological conditions as environmental variables. More precisely, for each fire regime considered, fire days represent the presences, whereas all days of the study period are the background. Daily values of the selected weather variable groups (meteo, indices, mixed) represent the explanatory environmental variables.

All model analyses were performed with the R statistical package, version 3.0.2 [48]. For the Maxent model, we used the ‘dismo’ R package (version 0.8–17) with the Maxent default settings.

### Model performance metrics

Model performances were evaluated with the widely-used metric AUC, which is the area under the ROC (relative operating characteristic) curve. The ROC graph is a way to depict classifiers’ ability (performance) to distinguish between correct and false classifications. ROC performances are frequently reduced to the single one-dimensional value Area Under the Curve (AUC), in order to enable a direct comparison among classifiers [49], [50], even if the single AUC value does not often communicate the rich information that the entire ROC curve can reveal [51].

The AUC is widely considered to be a very useful index (however, see [52] for a contrasting view) because it provides a single measure of overall accuracy that is not dependent upon a particular threshold [53], [54], [55]. The AUC value is obtained by calculating the area under the ROC curve derived by plotting the true positive fraction on the y-axis and the false positive fraction on the x-axis. It ranges from 0 to 1, where the 1 score indicates perfect discrimination, a 0.5 score implies predictive discrimination that is no better than a random guess, and values lower than 0.5 indicate performance worse than random. AUC is interpretable as the probability that a model discriminates correctly between presence and absence [56].

Where no reliable absence data is available, the AUC is calculated by distinguishing presence from the whole background (AUC.bg), or a random sub-sample, rather than presence from absence. This implies that the maximum possible AUC.bg will always be less than unity [57], [29]. If the species’ distribution covers a fraction *p* of the background (prevalence, see Table 2), then the prevalence-based theoretical maximum achievable AUC can be shown to be exactly 1 − *p/*2 [29] (see theoretical Maximum AUC.bg in Table 3). According to these assumptions, the AUC.bg is only suitable when comparing models trained on the same dataset. Our dataset consists of a reliable and small background allowing us to use the whole data for both modelling approaches.

A preliminary comparative test on the presence-absence logistic approach allowed us to show that the values resulting from the traditional AUC and from the AUC.bg were very similar (S1 Fig.). We therefore opted to use the AUC.bg to evaluate and make both the Maxent and logistic models comparable.

### Model evaluation

To evaluate the robustness of the selected models, a k-fold cross-validation [58], [59] was used in order to make the validation more meaningful and reliable [60]. The k-fold cross-validation consists in randomly splitting the whole original data into k subsets of equal size. Of the obtained k subsets, each one is used in turn for testing and the remaining k-1 subsets for training. In the splitting process, we paid particular attention to form folds (sub-periods) of two entire and consecutive years, obtaining 11 folds for w and sa (1991–2012) and 16 folds for sn (1981–2012) fire regimes.

The mean k-folds performances of the test cases were retained and used to compare the two model approaches. The differences between the classical logistic approach and Maxent were statistically verified using the non-parametric paired Wilcoxon Signed-rank test. We first tested the best models of the two modelling approaches, successively comparing the performance of both methods when applied to single indices. All statistical tests were run using the R statistical packages version 3.0.2 [48].

## Results

Table 3 reports the retained variables for the best Maxent models for each fire regime (w, sa, sn) and each group of explanatory variables considered (meteo, indices, mixed), as well as the related model performances in terms of mean, minimum, and maximum AUC.bg values reached by the k-folds cross-validations with respect to the theoretical Maximum AUC.bg. The number of explanatory variables retained ranges from 4 (indices w) to 7 (meteo and mixed w). The mean AUC.bg values for the three different groups of explanatory variables were rather high, ranging between 0.7516 (meteo sa) and 0.8517 (mixed sn). Absolute minimum test values resulted for k-folds models of meteo sa (AUC.bg = 0.5829 out of a theoretical maximum value of 0.9827), whereas the highest score was reached by the mixed sn (AUC.bg = 0.9760 out of a theoretical maximum value of 0.9890). When relating the results to the theoretical Maximum AUC.bg for each fire regime, the sa displays the lowest scores for every group of explanatory variables considered.

Comparing the results between the best Maxent and logistic models (see S1 Table for more details), most of the mean AUC.bg values for Maxent models were higher than those from logistic models, while the number of the selected variables was lower.

**Fig 3:**
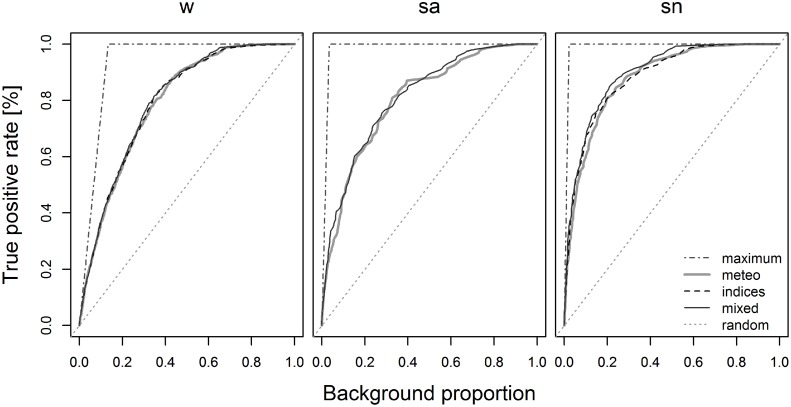
AUC.bg curves of the best Maxent models for the considered fire regimes (w, sa, sn). The prevalence-based theoretical Maximum AUC.bg lines are represented in dashed-dotted (w = 0.933, sa = 0.9827, sn = 0.9890) while the random guess lines (values of 0.5) are indicated using dotted lines. The Y-axis reports the true positive rate (%), that is the proportion of the fire days considered, while the X-axis indicates the background (total number of days) proportion.

Fig. 3 shows the plots of ROC curves of the best Maxent models with the theoretical Maximum AUC.bg curves and the random guess lines for all fire regimes. These plots relate the proportion of fire days predicted (the true positive rate) and the proportion of total days considered (the background proportion), highlighting the differences in the shape of the curves in comparison to the theoretical Maximum AUC.bg, as well as the better performances of mixed-variable models with respect to all others.

**Fig 4:**
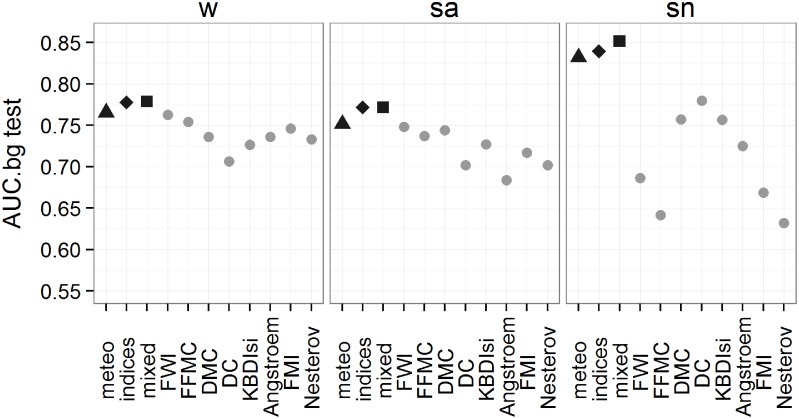
The mean k-fold performances of the test cases. Mean performance values of the best Maxent models (black) for the variable combinations compared to the logistic models (grey) of single indices for each fire regime.

Fig. 4 shows the performances of the Maxent models with respect to the logistic models of single selected indices. The best Maxent models derived from a combination of variables (meteo, indices and mixed) show higher AUC.bg values than the single index based on logistic models throughout, with greater differences for the sn fire regime. The values of the single index models range from 0.67 to 0.76, with the highest value for FWI in w and sa fire regimes and the DC in the sn regime. Similar results are found when comparing single indices and best models for both logistic and Maxent with higher values for Maxent models derived from combination of variables (see S2 Fig. for details).

The better performances of the best Maxent compared to logistic models are confirmed when selecting the first 10 best models for each case and each regime: all best models are Maxent according to each combination of variables (meteo, indices and mixed) for all regimes except for w and sa of meteo variables (S2 Table).

Most of the differences in AUC.bg shown in Fig. 4 (and S2 Fig.) are highly significant (Table 4 and S3 Table). The statistical significance is particularly clear in w and sn, in which all logistic models based on single indices are statistically different from the best Maxent models based on combinations of variables. A single exception exists regarding the FWI and FFMC in w compared to the best meteo model (Table 4). A slightly different situation exists with regards to the sa regime in which no statistical significance is reported for most of the indices compared with the best Maxent meteo model and the FWI and FFMC for all groups of explanatory variables.

**Table 4:** Results of the Wilcoxon rank sum tests among the best Maxent models and logistic models of single indices.

		w			sa			Sn		
		Maxent		
		meteo	indices	mixed	meteo	indices	mixed	meteo	indices	mixed
logistic	FWI	ns	**	**	ns	ns	ns	***	***	***
	FFMC	ns	**	*	ns	ns	ns	***	***	***
	DMC	**	***	***	ns	**	**	**	**	***
	DC	**	**	**	*	**	**	*	***	**
	KBDIsi	*	***	***	ns	*	*	*	***	***
	Angstroem	**	***	***	*	*	*	**	***	***
	FMI	*	***	**	ns	*	*	***	***	***
	Nesterov	***	***	***	*	**	**	***	***	***

*** = p <0.001

** = p <0.01

* = p <0.05, ns = not significant.

Similar results emerge from the comparison of the best logistic with the best Maxent models with combinations of variables and single indices models based on the two different approaches (see S3 Table).

The comparison of the best models (Table 5) reveals that Maxent meteo models are significantly different only from the best logistic meteo model for sn, while the best Maxent indices models are statistically different from the best logistic meteo and mixed models in w and from the best logistic indices model in sa. Finally, the best mixed Maxent models show significant differences with respect to the best logistic meteo model for w and sn, and the best logistic indices model for sa (see S4 Table for more details).

**Table 5:** Results of the Wilcoxon rank sum tests among the best Maxent and logistic models.

		w			sa			sn		
		Maxent								
		meteo	indices	mixed	meteo	indices	mixed	meteo	indices	mixed
logistic	meteo	Ns	*	*	ns	ns	ns	*	ns	*
	indices	ns	ns	ns	ns	*	*	ns	ns	ns
	mixed	ns	*	ns	ns	ns	ns	ns	ns	ns

* = p <0.05, ns = not significant.

## Discussion

The aim of this study was to develop and test a method for developing a new fire weather danger ratings approach. To this purpose we modelled the fire occurrences in homogeneous fire regime conditions in Ticino, a low to intermediate fire prone region in the Swiss Alps using meteorological parameters, fire weather indices or a combination of both as inputs. Our results highlight that considering fire occurrence as response variables and combining meteorological-based input parameters allows a better characterization of the meteorological “fire niche” and increases the performance and reliability of the related predictive models.

When selecting variables from different pools (meteo and indices) to build models, the predictive power is additionally enhanced. Interestingly, also the option of using only meteorological variables allows us to obtain performances equivalent to the Canadian FWI in w and sa regimes, and much higher than every single index in sn, opening new perspectives for cases where the indices can not be calculated.

The modelling approach based on Maxent resulted in slightly improved model performance and a reduction in the number of variables selected in the best models with respect to the classical logistic approach. Elith et al. [30] have also come to similar conclusions, demonstrating that Maxent models outperform logistic regressions fitted using generalised linear models when dealing with species distribution studies. The better predictive power of Maxent compared to the generalized linear model is also reported by other authors [61], [62], [63], [64], [65]. Bar Massada et al. [34] in particular have already applied a Maxent approach to the field of forest fires, highlighting the advantages of the Maxent approach over logistic models when dealing with fire ignition distribution modelling.

The best Maxent models were also found to be reliable and robust as demonstrated by mean performance values greater than 0.75 and the low variability among the folds of the k-fold cross-validation. When looking at the different fire regimes, it becomes evident that factors influencing final model robustness are basically the number of fire events available and the specificity of the meteorological conditions leading to fire ignition. Thus, for instance, the w fire regime enabled the development of a robust model (see performance range of k-folds AUC.bg in Table 3) thanks to the fairly high number of fires available and the quite specific meteorological characteristics when fires occur, often related to foehn conditions and mainly in the months of March and April (70% of the w fires). In case of lightning fires, model robustness mostly depends on the very high specificity and the absolutely dominant influence of meteorological conditions at the time of fire ignition, specifically the dry preceding period and thunderstorms without heavy rainfall at the moment of ignition (77.6% of the sn fires are in July and August). Human activities have only a very indirect influence indeed, in terms of fuel build up as a function of land use in case of lightning-induced fires [39], [66]. Less efficient models resulted for the sa regime because of both the low number of fires available and the unspecific characteristics of the human-induced fire ignitions during the hot summer period. As a result, the relative performance values are lower than in other regimes and its range is very broad.

The presented method is very flexible and can be easily applied also in more fire prone regions displaying high fire occurrences and a significant number of large fires by setting for instance the criterion of a minimum of area burnt to assign the presence attribute to a fire day. Doing so, you turn the fire danger rating into a system for predicting any “target-size” fire days, so to adapt it to the local operational needs.

## Conclusion

The described approach combines a number of variables for identifying the daily suitable meteorological conditions for fires. However, while the resulting index fits best the meteorological and pyrological characteristics of the region under analysis, it is not directly applicable to different regions. For this reason the proposed approach needs to be applied for every region of interest, assuming that suitable historical fire data and detailed and consistent meteorological data are available. Our approach also implies the need to update the model selection, and therefore the index calculation, in case of changes in the pyrological environment under consideration, specifically new legislation and significant socio-economic changes. Thanks to the fire niche approach, however, acceptable fire weather indices can be developed and operationally run starting from fairly small fire numbers, which is often the case in low-to-moderate fire prone regions. In case of fire prone regions with high fire occurrence and a significant number of large fires, the present rating approach should be adapted to the local operational needs, considering for example only fires with a minimum area in order to predict large or target-size fire days.

## Supporting Information

S1 Fig Mean AUC.bg and AUC values of the test cases for the logistic best models and the w, sa and sn fire regimes. The upper row represents the best models according to the AUC.bg.test, while the lower refers to the AUC.test. Symbols refer to the different combination of variables: circle for meteo, triangle for indices and square for mixed.
(DOC)

S2 Fig The mean performance values of the test cases for each fire regime. The plots show the comparison between the Maxent (empty black symbols) and the logistic models (full grey symbols) for all variable combinations and single indices for w, sa and sn regimes.
(DOC)

S1 Table Best models for the logistic and Maxent modelling approaches. Variable selection and mean AUC.bg value of the test cases of the k-folds are reported for each best model.
(DOC)

S2 Table The ten best models for each regime and combination of variables. (DOC)

S3 Table Results of the Wilcoxon rank sum tests for the best and the single indices models. The results are referred to the comparison between the best models based on the different variables combination and the models on the single indices using the logistic (a) and Maxent (b) approaches.
(DOC)

S4 Table Results of the Wilcoxon rank sum tests for the best models. The results are referred to the comparison between the best models based on the different variables combination using the logistic (a) and Maxent (b) approaches.
(DOC)

S1 Data Dataset used for the analysis. (ZIP)
