# Supplementary material for: Modelling the Meteorological Forest Fire Niche in Heterogeneous Pyrologic Conditions
Source: PLoS One. 2015 Feb 13;10(2):e0116875. doi: 10.1371/journal.pone.0116875 (PMC4332634; doi:10.1371/journal.pone.0116875)
Supplement: S1 Fig — The upper row represents the best models according to the AUC.bg.test, while the lower refers to the AUC.test. Symbols refer to the different combination of variables: circle for meteo, triangle for indices and square for mixed. (DOC) [file pone.0116875.s001.doc]

**Figure S1.**

**Mean AUC.bg and AUC values of the test cases for the logistic best models and the w, sa and sn fire regimes.**

The upper row represents the best models according to the AUC.bg.test, while the lower refers to the AUC.test. Symbols refer to the different combination of variables: circle for meteo, triangle for indices and square for mixed.
