# Supplementary material for: Modelling the Meteorological Forest Fire Niche in Heterogeneous Pyrologic Conditions
Source: PLoS One. 2015 Feb 13;10(2):e0116875. doi: 10.1371/journal.pone.0116875 (PMC4332634; doi:10.1371/journal.pone.0116875)
Supplement: S2 Fig — The plots show the comparison between the Maxent (empty black symbols) and the logistic models (full grey symbols) for all variable combinations and single indices for w, sa and sn regimes. (DOC) [file pone.0116875.s002.doc]

**Figure S2.**

**The mean performance values of the test cases for each fire regime.**

The plots show the comparison between the Maxent (empty black symbols) and the logistic models (full grey symbols) for all variable combinations and single indices for w, sa and sn regimes.
