# Supplementary material for: Modelling the Meteorological Forest Fire Niche in Heterogeneous Pyrologic Conditions
Source: PLoS One. 2015 Feb 13;10(2):e0116875. doi: 10.1371/journal.pone.0116875 (PMC4332634; doi:10.1371/journal.pone.0116875)
Supplement: S1 Table — Variable selection and mean AUC.bg value of the test cases of the k-folds are reported for each best model. (DOC) [file pone.0116875.s003.doc]

**Table S1.**

**Best models for the logistic and Maxent modelling approaches.**

Variable selection and mean AUC.bg value of the test cases of the k-folds are reported for each best model.

| **REGIME** | **BEST MODELS** |  | **Input meteorological variables** | | | | | | | | | | | **Input indices** | | | | | | | | | | | | | | | **Performances** |
| --- | --- | --- | --- | --- | --- | --- | --- | --- | --- | --- | --- | --- | --- | --- | --- | --- | --- | --- | --- | --- | --- | --- | --- | --- | --- | --- | --- | --- | --- |
|  |  |  | **T [°C]** | **Tdew [°C]** | **P [mm]** | **U [m/s]** | **H [%]** | **VPD [kPa]** | **CloudCover [ratio 0/1]** | **Weekrain [mm/week]** | **LastRainSum [mm]** | **DaysSinceRain [day]** | **SnowCover [0/1]** | **Angstroem** | **Baumgartner** | **FFMC** | **DMC** | **DC** | **ISI** | **BUI** | **FWI** | **FFWI** | **KBDIsi** | **FFDI** | **Munger** | **Orieuxdanger** | **Nesterov** | **FMI** | **Mean Auc.bg test** |
| **w** | **meteo** | logistic | ○ | ● | ● | ○ | ● | ○ | ● | ● | ○ | ● | ● |  |  |  |  |  |  |  |  |  |  |  |  |  |  |  | 0.7676 |
|  |  | Maxent | ○ | ● | ● | ○ | ● | ○ | ● | ● | ● | ○ | ● |  |  |  |  |  |  |  |  |  |  |  |  |  |  |  | 0.7655 |
|  | **indices** | logistic |  |  |  |  |  |  |  |  |  |  |  | ○ | ● | ● | ○ | ○ | ○ | ● | ○ | ○ | ● | ○ | ● | ● | ● | ● | 0.7730 |
|  |  | Maxent |  |  |  |  |  |  |  |  |  |  |  | ○ | ○ | ○ | ● | ○ | ○ | ○ | ○ | ○ | ○ | ○ | ○ | ● | ● | ● | 0.7776 |
|  | **mixed** | logistic | ○ | ● | ● | ○ | ○ |  | ● | ○ |  |  | ● |  | ● |  | ○ |  |  |  | ○ | ○ |  | ● | ● | ● | ● | ● | 0.7721 |
|  |  | Maxent | ○ | ○ | ● | ○ | ○ |  | ● | ○ |  |  | ● |  | ○ |  | ● |  |  |  | ○ | ○ |  | ○ | ○ | ● | ● | ● | 0.7789 |
| **sa** | **meteo** | logistic | ● | ○ | ○ | ● | ○ | ● | ● | ● | ○ | ● |  |  |  |  |  |  |  |  |  |  |  |  |  |  |  |  | 0.7548 |
|  |  | Maxent | ● | ○ | ○ | ● | ○ | ● | ○ | ● | ○ | ● |  |  |  |  |  |  |  |  |  |  |  |  |  |  |  |  | 0.7516 |
|  | **indices** | logistic |  |  |  |  |  |  |  |  |  |  |  | ● | ○ | ● | ○ | ○ | ○ | ○ | ○ | ● | ● | ● | ● | ● | ○ | ○ | 0.7615 |
|  |  | Maxent |  |  |  |  |  |  |  |  |  |  |  | ○ | ○ | ○ | ● | ○ | ○ | ○ | ○ | ● | ○ | ● | ● | ● | ○ | ○ | 0.7716 |
|  | **mixed** | logistic | ○ | ○ | ○ | ○ | ● |  | ○ | ● |  |  |  |  | ○ |  | ● |  |  |  | ○ | ○ |  | ● | ● | ○ | ○ | ○ | 0.7586 |
|  |  | Maxent | ○ | ○ | ○ | ○ | ○ |  | ○ | ○ |  |  |  |  | ○ |  | ● |  |  |  | ○ | ● |  | ● | ● | ● | ○ | ○ | 0.7716 |
| **sn** | **meteo** | logistic | ○ | ● | ● | ● | ○ | ● | ● | ● | ● | ○ |  |  |  |  |  |  |  |  |  |  |  |  |  |  |  |  | 0.8208 |
|  |  | Maxent | ○ | ○ | ○ | ● | ● | ● | ● | ● | ○ | ● |  |  |  |  |  |  |  |  |  |  |  |  |  |  |  |  | 0.8320 |
|  | **indices** | logistic |  |  |  |  |  |  |  |  |  |  |  | ● | ● | ● | ● | ● | ○ | ○ | ○ | ○ | ○ | ● | ● | ○ | ○ | ○ | 0.8274 |
|  |  | Maxent |  |  |  |  |  |  |  |  |  |  |  | ○ | ● | ○ | ○ | ○ | ○ | ○ | ● | ○ | ○ | ○ | ● | ● | ○ | ● | 0.8393 |
|  | **mixed** | logistic | ○ | ● | ○ | ○ | ○ |  | ○ | ○ |  |  |  |  | ● |  | ● |  |  |  | ● | ● |  | ● | ● | ● | ○ | ○ | 0.8310 |
|  |  | Maxent | ○ | ● | ○ | ● | ● |  | ○ | ○ |  |  |  |  | ● |  | ○ |  |  |  | ● | ○ |  | ○ | ● | ● | ○ | ○ | 0.8517 |
