# Supplementary material for: Modelling the Meteorological Forest Fire Niche in Heterogeneous Pyrologic Conditions
Source: PLoS One. 2015 Feb 13;10(2):e0116875. doi: 10.1371/journal.pone.0116875 (PMC4332634; doi:10.1371/journal.pone.0116875)
Supplement: S2 Table — (DOC) [file pone.0116875.s004.doc]

**Table S2.**

**The ten best models for each regime** and combination of variables.

| **REGIME** | **BEST MODELS** | **Model approaches** | **Input meteorological variables** | | | | | | | | | | | **Input indices** | | | | | | | | | | | | | | | **Performances** | |
| --- | --- | --- | --- | --- | --- | --- | --- | --- | --- | --- | --- | --- | --- | --- | --- | --- | --- | --- | --- | --- | --- | --- | --- | --- | --- | --- | --- | --- | --- | --- |
|  |  |  | **T [°C]** | **Tdew [°C]** | **P [mm]** | **U [m/s]** | **H [%]** | **VPD [kPa]** | **CloudCover**  **[ratio 0/1]** | **Weekrain [mm/week]** | **LastRainSum [mm]** | **DaysSinceRain [day]** | **SnowCover [0/1]** | **Angstroem** | **Baumgartner** | **FFMC** | **DMC** | **DC** | **ISI** | **BUI** | **FWI** | **FFWI** | **KBDIsi** | **FFDI** | **Munger** | **Orieuxdanger** | **Nesterov** | **FMI** | | **Mean Auc.bg test** |
| **w** | **meteo** | **logistic** | ○ | ● | ● | ○ | ● | ○ | ● | ● | ○ | ● | ● |  |  |  |  |  |  |  |  |  |  |  |  |  |  |  | | 0.7676 |
|  |  |  | ● | ● | ● | ○ | ○ | ○ | ● | ● | ○ | ● | ● |  |  |  |  |  |  |  |  |  |  |  |  |  |  |  | | 0.7673 |
|  |  |  | ● | ○ | ● | ○ | ● | ○ | ● | ● | ○ | ● | ● |  |  |  |  |  |  |  |  |  |  |  |  |  |  |  | | 0.7673 |
|  |  |  | ● | ● | ● | ○ | ● | ○ | ● | ● | ○ | ● | ● |  |  |  |  |  |  |  |  |  |  |  |  |  |  |  | | 0.7673 |
|  |  |  | ○ | ● | ● | ○ | ● | ○ | ● | ● | ● | ● | ● |  |  |  |  |  |  |  |  |  |  |  |  |  |  |  | | 0.7672 |
|  |  |  | ● | ○ | ● | ○ | ● | ○ | ● | ● | ● | ● | ● |  |  |  |  |  |  |  |  |  |  |  |  |  |  |  | | 0.7671 |
|  |  |  | ● | ● | ● | ○ | ● | ○ | ● | ● | ● | ● | ● |  |  |  |  |  |  |  |  |  |  |  |  |  |  |  | | 0.7671 |
|  |  |  | ● | ● | ● | ○ | ○ | ○ | ● | ● | ● | ● | ● |  |  |  |  |  |  |  |  |  |  |  |  |  |  |  | | 0.7670 |
|  |  |  | ○ | ○ | ● | ○ | ○ | ● | ○ | ● | ○ | ● | ● |  |  |  |  |  |  |  |  |  |  |  |  |  |  |  | | 0.7670 |
|  |  |  | ○ | ○ | ● | ○ | ○ | ● | ● | ● | ○ | ● | ● |  |  |  |  |  |  |  |  |  |  |  |  |  |  |  | | 0.7670 |
|  | **indices** | **Maxent** |  |  |  |  |  |  |  |  |  |  |  | ○ | ○ | ○ | ● | ○ | ○ | ○ | ○ | ○ | ○ | ○ | ○ | ● | ● | ● | | 0.7548 |
|  |  |  |  |  |  |  |  |  |  |  |  |  |  | ○ | ○ | ○ | ● | ○ | ○ | ○ | ○ | ○ | ○ | ○ | ○ | ○ | ● | ● | | 0.7546 |
|  |  |  |  |  |  |  |  |  |  |  |  |  |  | ○ | ○ | ○ | ○ | ○ | ○ | ● | ○ | ○ | ○ | ○ | ○ | ● | ● | ● | | 0.7544 |
|  |  |  |  |  |  |  |  |  |  |  |  |  |  | ○ | ○ | ○ | ● | ○ | ○ | ○ | ○ | ○ | ○ | ● | ○ | ○ | ● | ● | | 0.7544 |
|  |  |  |  |  |  |  |  |  |  |  |  |  |  | ○ | ● | ○ | ● | ○ | ○ | ○ | ○ | ○ | ○ | ○ | ○ | ● | ● | ● | | 0.7543 |
|  |  |  |  |  |  |  |  |  |  |  |  |  |  | ○ | ○ | ○ | ○ | ○ | ○ | ● | ○ | ○ | ○ | ○ | ○ | ○ | ● | ● | | 0.7541 |
|  |  |  |  |  |  |  |  |  |  |  |  |  |  | ○ | ● | ○ | ● | ○ | ○ | ○ | ○ | ○ | ○ | ○ | ○ | ○ | ● | ● | | 0.7540 |
|  |  |  |  |  |  |  |  |  |  |  |  |  |  | ○ | ○ | ○ | ○ | ○ | ○ | ● | ○ | ○ | ○ | ● | ○ | ● | ● | ● | | 0.7540 |
|  |  |  |  |  |  |  |  |  |  |  |  |  |  | ○ | ○ | ○ | ● | ○ | ○ | ○ | ○ | ○ | ○ | ● | ○ | ○ | ● | ● | | 0.7540 |
|  |  |  |  |  |  |  |  |  |  |  |  |  |  | ○ | ● | ○ | ○ | ○ | ○ | ● | ○ | ○ | ○ | ○ | ○ | ● | ● | ● | | 0.7540 |
|  | **mixed** | **Maxent** | ○ | ○ | ● | ○ | ○ | ○ | ● | ○ | ○ | ○ | ● | ○ | ○ | ○ | ● | ○ | ○ | ○ | ○ | ○ | ○ | ○ | ○ | ● | ● | ● | | 0.8320 |
|  |  |  | ○ | ○ | ● | ○ | ○ | ○ | ● | ○ | ○ | ○ | ○ | ○ | ○ | ○ | ● | ○ | ○ | ○ | ○ | ○ | ○ | ○ | ○ | ● | ● | ● | | 0.8314 |
|  |  |  | ○ | ○ | ● | ○ | ○ | ○ | ● | ○ | ○ | ○ | ● | ○ | ○ | ○ | ● | ○ | ○ | ○ | ○ | ○ | ○ | ○ | ○ | ○ | ● | ● | | 0.8314 |
|  |  |  | ○ | ○ | ● | ○ | ○ | ○ | ● | ○ | ○ | ○ | ○ | ○ | ○ | ○ | ● | ○ | ○ | ○ | ○ | ○ | ○ | ● | ○ | ● | ● | ● | | 0.8308 |
|  |  |  | ○ | ○ | ● | ○ | ○ | ○ | ● | ○ | ○ | ○ | ● | ○ | ○ | ○ | ● | ○ | ○ | ○ | ○ | ○ | ○ | ● | ○ | ● | ● | ● | | 0.8308 |
|  |  |  | ○ | ○ | ● | ○ | ○ | ○ | ● | ○ | ○ | ○ | ○ | ○ | ○ | ○ | ● | ○ | ○ | ○ | ○ | ○ | ○ | ○ | ○ | ○ | ● | ● | | 0.8307 |
|  |  |  | ○ | ○ | ● | ○ | ○ | ○ | ● | ○ | ○ | ○ | ● | ○ | ○ | ○ | ● | ○ | ○ | ○ | ○ | ○ | ○ | ● | ○ | ○ | ● | ● | | 0.8303 |
|  |  |  | ○ | ○ | ● | ○ | ● | ○ | ● | ○ | ○ | ○ | ● | ○ | ○ | ○ | ● | ○ | ○ | ○ | ○ | ○ | ○ | ● | ○ | ● | ● | ○ | | 0.8301 |
|  |  |  | ○ | ○ | ● | ○ | ● | ○ | ● | ○ | ○ | ○ | ○ | ○ | ○ | ○ | ● | ○ | ○ | ○ | ○ | ○ | ○ | ● | ○ | ○ | ● | ○ | | 0.8301 |
|  |  |  | ○ | ○ | ○ | ○ | ○ | ○ | ● | ○ | ○ | ○ | ● | ○ | ○ | ○ | ● | ○ | ○ | ○ | ○ | ○ | ○ | ○ | ○ | ● | ● | ● | | 0.8298 |
| **sa** | **meteo** | **logistic** | ● | ○ | ○ | ● | ○ | ● | ● | ● | ○ | ● | ○ |  |  |  |  |  |  |  |  |  |  |  |  |  |  |  | | 0.7776 |
|  |  |  | ● | ○ | ○ | ● | ○ | ● | ○ | ● | ● | ● | ○ |  |  |  |  |  |  |  |  |  |  |  |  |  |  |  | | 0.7774 |
|  |  |  | ● | ○ | ○ | ● | ○ | ● | ● | ● | ● | ● | ○ |  |  |  |  |  |  |  |  |  |  |  |  |  |  |  | | 0.7771 |
|  |  |  | ● | ○ | ○ | ● | ○ | ● | ○ | ● | ○ | ● | ○ |  |  |  |  |  |  |  |  |  |  |  |  |  |  |  | | 0.7771 |
|  |  |  | ● | ● | ○ | ● | ● | ● | ● | ● | ○ | ● | ○ |  |  |  |  |  |  |  |  |  |  |  |  |  |  |  | | 0.7770 |
|  |  |  | ● | ● | ○ | ● | ○ | ● | ○ | ● | ● | ● | ○ |  |  |  |  |  |  |  |  |  |  |  |  |  |  |  | | 0.7769 |
|  |  |  | ○ | ● | ○ | ● | ○ | ● | ● | ● | ○ | ● | ○ |  |  |  |  |  |  |  |  |  |  |  |  |  |  |  | | 0.7768 |
|  |  |  | ○ | ● | ○ | ● | ○ | ● | ● | ● | ● | ● | ○ |  |  |  |  |  |  |  |  |  |  |  |  |  |  |  | | 0.7768 |
|  |  |  | ● | ● | ○ | ● | ○ | ● | ● | ● | ● | ● | ○ |  |  |  |  |  |  |  |  |  |  |  |  |  |  |  | | 0.7767 |
|  |  |  | ● | ● | ○ | ● | ○ | ● | ○ | ● | ○ | ● | ○ |  |  |  |  |  |  |  |  |  |  |  |  |  |  |  | | 0.7765 |
|  | **indices** | **Maxent** |  |  |  |  |  |  |  |  |  |  |  | ○ | ○ | ○ | ● | ○ | ○ | ○ | ○ | ● | ○ | ● | ● | ● | ○ | ○ | | 0.7716 |
|  |  |  |  |  |  |  |  |  |  |  |  |  |  | ○ | ○ | ○ | ● | ○ | ○ | ○ | ○ | ● | ○ | ○ | ● | ● | ○ | ○ | | 0.7710 |
|  |  |  |  |  |  |  |  |  |  |  |  |  |  | ○ | ○ | ○ | ● | ● | ○ | ○ | ○ | ● | ○ | ● | ● | ● | ○ | ○ | | 0.7693 |
|  |  |  |  |  |  |  |  |  |  |  |  |  |  | ○ | ○ | ○ | ● | ● | ○ | ○ | ○ | ● | ○ | ○ | ● | ● | ○ | ○ | | 0.7691 |
|  |  |  |  |  |  |  |  |  |  |  |  |  |  | ○ | ○ | ○ | ○ | ● | ○ | ○ | ○ | ● | ○ | ● | ● | ● | ○ | ○ | | 0.7689 |
|  |  |  |  |  |  |  |  |  |  |  |  |  |  | ○ | ○ | ○ | ● | ○ | ○ | ● | ○ | ● | ○ | ● | ● | ● | ○ | ○ | | 0.7687 |
|  |  |  |  |  |  |  |  |  |  |  |  |  |  | ○ | ○ | ○ | ● | ○ | ● | ○ | ○ | ● | ○ | ● | ● | ● | ○ | ○ | | 0.7687 |
|  |  |  |  |  |  |  |  |  |  |  |  |  |  | ○ | ○ | ○ | ● | ● | ● | ○ | ○ | ● | ○ | ● | ● | ● | ○ | ○ | | 0.7686 |
|  |  |  |  |  |  |  |  |  |  |  |  |  |  | ○ | ○ | ● | ● | ● | ○ | ○ | ○ | ● | ○ | ● | ● | ● | ○ | ○ | | 0.7686 |
|  |  |  |  |  |  |  |  |  |  |  |  |  |  | ○ | ○ | ○ | ● | ● | ○ | ○ | ○ | ● | ○ | ● | ● | ○ | ○ | ○ | | 0.7683 |
|  | **mixed** | **Maxent** | ○ | ○ | ○ | ○ | ○ | ○ | ○ | ○ | ○ | ○ | ○ | ○ | ○ | ○ | ● | ○ | ○ | ○ | ○ | ● | ○ | ● | ● | ● | ○ | ○ | | 0.8393 |
|  |  |  | ● | ○ | ○ | ● | ○ | ○ | ○ | ○ | ○ | ○ | ○ | ○ | ○ | ○ | ● | ○ | ○ | ○ | ○ | ○ | ○ | ● | ○ | ○ | ○ | ○ | | 0.8386 |
|  |  |  | ○ | ○ | ○ | ○ | ○ | ○ | ○ | ○ | ○ | ○ | ○ | ○ | ○ | ○ | ● | ○ | ○ | ○ | ○ | ● | ○ | ○ | ● | ● | ○ | ○ | | 0.8373 |
|  |  |  | ● | ○ | ○ | ○ | ○ | ○ | ○ | ○ | ○ | ○ | ○ | ○ | ○ | ○ | ● | ○ | ○ | ○ | ○ | ● | ○ | ● | ○ | ● | ○ | ○ | | 0.8363 |
|  |  |  | ● | ○ | ○ | ○ | ● | ○ | ○ | ○ | ○ | ○ | ○ | ○ | ○ | ○ | ● | ○ | ○ | ○ | ○ | ○ | ○ | ● | ○ | ● | ○ | ○ | | 0.8360 |
|  |  |  | ● | ○ | ○ | ○ | ○ | ○ | ○ | ○ | ○ | ○ | ○ | ○ | ○ | ○ | ● | ○ | ○ | ○ | ○ | ● | ○ | ○ | ○ | ○ | ○ | ○ | | 0.8359 |
|  |  |  | ● | ○ | ○ | ○ | ○ | ○ | ○ | ○ | ○ | ○ | ○ | ○ | ○ | ○ | ● | ○ | ○ | ○ | ○ | ● | ○ | ● | ○ | ○ | ○ | ○ | | 0.8359 |
|  |  |  | ● | ○ | ○ | ● | ○ | ○ | ○ | ○ | ○ | ○ | ○ | ○ | ○ | ○ | ● | ○ | ○ | ○ | ○ | ○ | ○ | ● | ○ | ● | ○ | ○ | | 0.8356 |
|  |  |  | ● | ○ | ○ | ○ | ○ | ○ | ○ | ○ | ○ | ○ | ○ | ○ | ○ | ○ | ● | ○ | ○ | ○ | ○ | ● | ○ | ● | ○ | ● | ● | ○ | | 0.8356 |
|  |  |  | ○ | ○ | ○ | ● | ○ | ○ | ○ | ○ | ○ | ○ | ○ | ○ | ○ | ○ | ● | ○ | ○ | ○ | ○ | ○ | ○ | ● | ● | ● | ○ | ○ | | 0.8349 |
| **sn** | **meteo** | **Maxent** | ○ | ○ | ○ | ● | ● | ● | ● | ● | ● | ○ | ○ |  |  |  |  |  |  |  |  |  |  |  |  |  |  |  | | 0.7789 |
|  |  |  | ● | ○ | ● | ○ | ● | ● | ○ | ● | ● | ○ | ○ |  |  |  |  |  |  |  |  |  |  |  |  |  |  |  | | 0.7789 |
|  |  |  | ○ | ○ | ● | ● | ● | ● | ○ | ● | ● | ○ | ○ |  |  |  |  |  |  |  |  |  |  |  |  |  |  |  | | 0.7787 |
|  |  |  | ○ | ○ | ● | ● | ● | ● | ● | ● | ● | ○ | ○ |  |  |  |  |  |  |  |  |  |  |  |  |  |  |  | | 0.7786 |
|  |  |  | ○ | ○ | ○ | ● | ● | ● | ○ | ● | ● | ○ | ○ |  |  |  |  |  |  |  |  |  |  |  |  |  |  |  | | 0.7786 |
|  |  |  | ○ | ● | ● | ● | ● | ● | ○ | ● | ● | ○ | ○ |  |  |  |  |  |  |  |  |  |  |  |  |  |  |  | | 0.7786 |
|  |  |  | ○ | ● | ○ | ● | ● | ● | ● | ● | ● | ○ | ○ |  |  |  |  |  |  |  |  |  |  |  |  |  |  |  | | 0.7785 |
|  |  |  | ● | ○ | ○ | ● | ● | ○ | ● | ● | ● | ○ | ○ |  |  |  |  |  |  |  |  |  |  |  |  |  |  |  | | 0.7784 |
|  |  |  | ● | ○ | ○ | ● | ● | ● | ○ | ● | ● | ○ | ○ |  |  |  |  |  |  |  |  |  |  |  |  |  |  |  | | 0.7784 |
|  |  |  | ● | ● | ● | ○ | ● | ● | ● | ● | ● | ○ | ○ |  |  |  |  |  |  |  |  |  |  |  |  |  |  |  | | 0.7784 |
|  | **indices** | **Maxent** |  |  |  |  |  |  |  |  |  |  |  | ○ | ● | ○ | ○ | ○ | ○ | ○ | ● | ○ | ○ | ○ | ● | ● | ○ | ● | | 0.7716 |
|  |  |  |  |  |  |  |  |  |  |  |  |  |  | ○ | ● | ○ | ○ | ○ | ○ | ○ | ● | ○ | ○ | ● | ● | ● | ○ | ● | | 0.7711 |
|  |  |  |  |  |  |  |  |  |  |  |  |  |  | ○ | ● | ○ | ○ | ○ | ○ | ○ | ● | ○ | ○ | ● | ● | ● | ● | ● | | 0.7710 |
|  |  |  |  |  |  |  |  |  |  |  |  |  |  | ● | ● | ○ | ● | ○ | ○ | ○ | ● | ○ | ○ | ● | ● | ○ | ○ | ○ | | 0.7708 |
|  |  |  |  |  |  |  |  |  |  |  |  |  |  | ○ | ● | ○ | ● | ○ | ○ | ○ | ● | ○ | ○ | ● | ● | ● | ○ | ● | | 0.7703 |
|  |  |  |  |  |  |  |  |  |  |  |  |  |  | ○ | ● | ○ | ○ | ○ | ○ | ○ | ● | ○ | ○ | ○ | ● | ● | ● | ● | | 0.7698 |
|  |  |  |  |  |  |  |  |  |  |  |  |  |  | ● | ● | ○ | ● | ○ | ○ | ○ | ● | ● | ○ | ● | ● | ○ | ○ | ○ | | 0.7695 |
|  |  |  |  |  |  |  |  |  |  |  |  |  |  | ● | ● | ● | ● | ○ | ○ | ○ | ○ | ● | ○ | ○ | ● | ○ | ○ | ○ | | 0.7695 |
|  |  |  |  |  |  |  |  |  |  |  |  |  |  | ○ | ● | ○ | ● | ○ | ○ | ○ | ○ | ○ | ○ | ● | ● | ● | ○ | ● | | 0.7694 |
|  |  |  |  |  |  |  |  |  |  |  |  |  |  | ○ | ● | ○ | ● | ○ | ○ | ○ | ● | ● | ○ | ● | ● | ○ | ○ | ○ | | 0.7693 |
|  | **mixed** | **Maxent** | ○ | ● | ○ | ● | ● | ○ | ○ | ○ | ○ | ○ | ○ | ○ | ● | ○ | ○ | ○ | ○ | ○ | ● | ○ | ○ | ○ | ● | ● | ○ | ○ | | 0.8517 |
|  |  |  | ○ | ● | ○ | ● | ● | ○ | ○ | ○ | ○ | ○ | ○ | ○ | ● | ○ | ○ | ○ | ○ | ○ | ● | ● | ○ | ○ | ● | ● | ○ | ○ | | 0.8515 |
|  |  |  | ○ | ● | ○ | ● | ● | ○ | ○ | ○ | ○ | ○ | ○ | ○ | ● | ○ | ○ | ○ | ○ | ○ | ● | ● | ○ | ○ | ● | ● | ○ | ○ | | 0.8506 |
|  |  |  | ○ | ● | ○ | ● | ● | ○ | ○ | ○ | ○ | ○ | ○ | ○ | ● | ○ | ○ | ○ | ○ | ○ | ● | ● | ○ | ● | ● | ● | ○ | ○ | | 0.8505 |
|  |  |  | ○ | ● | ○ | ○ | ● | ○ | ○ | ○ | ○ | ○ | ○ | ○ | ● | ○ | ○ | ○ | ○ | ○ | ● | ● | ○ | ○ | ● | ● | ○ | ○ | | 0.8497 |
|  |  |  | ○ | ● | ○ | ● | ● | ○ | ○ | ○ | ○ | ○ | ○ | ○ | ● | ○ | ○ | ○ | ○ | ○ | ● | ● | ○ | ● | ● | ● | ● | ○ | | 0.8495 |
|  |  |  | ○ | ● | ○ | ● | ● | ○ | ● | ○ | ○ | ○ | ○ | ○ | ● | ○ | ○ | ○ | ○ | ○ | ● | ○ | ○ | ● | ● | ● | ○ | ○ | | 0.8494 |
|  |  |  | ○ | ● | ○ | ○ | ● | ○ | ○ | ○ | ○ | ○ | ○ | ○ | ● | ○ | ○ | ○ | ○ | ○ | ● | ○ | ○ | ● | ● | ● | ○ | ○ | | 0.8493 |
|  |  |  | ○ | ● | ○ | ● | ● | ○ | ○ | ○ | ○ | ○ | ○ | ○ | ● | ○ | ○ | ○ | ○ | ○ | ● | ● | ○ | ○ | ● | ● | ● | ○ | | 0.8493 |
|  |  |  | ○ | ● | ● | ● | ● | ○ | ○ | ○ | ○ | ○ | ○ | ○ | ● | ○ | ○ | ○ | ○ | ○ | ● | ○ | ○ | ● | ● | ● | ○ | ○ | | 0.8489 |
