# Supplementary material for: Modelling the Meteorological Forest Fire Niche in Heterogeneous Pyrologic Conditions
Source: PLoS One. 2015 Feb 13;10(2):e0116875. doi: 10.1371/journal.pone.0116875 (PMC4332634; doi:10.1371/journal.pone.0116875)
Supplement: S3 Table — The results are referred to the comparison between the best models based on the different variables combination and the models on the single indices using the logistic (a) and Maxent (b) approaches. (DOC) [file pone.0116875.s005.doc]

**Table S3.**

**Results of the Wilcoxon rank sum tests for the best and the single indices models.**

The results are referred to the comparison between the best models based on the different variables combination and the models on the single indices using the logistic (a) and Maxent (b) approaches.

(a) logistic

|  | w | | | sa | | | sn | | |
| --- | --- | --- | --- | --- | --- | --- | --- | --- | --- |
|  | meteo | indices | mixed | meteo | indices | mixed | meteo | indices | mixed |
| FWI | ns | *** | ns | ns | ns | ns | *** | *** | ** |
| FFMC | ns | * | * | ns | ns | ns | *** | *** | *** |
| DMC | ** | ** | ** | ns | * | ns | ** | ** | ** |
| DC | ** | ** | ** | * | ** | * | * | * | ** |
| KBDIsi | ** | ** | ** | ns | ns | ns | * | ** | *** |
| Angstroem | ** | *** | *** | * | * | * | ** | ** | *** |
| FMI | ** | *** | ** | ns | * | ns | *** | *** | *** |
| Nesterov | ** | *** | *** | * | * | * | *** | *** | ** |

(b) Maxent

|  | w | | | sa | | | sn | | |
| --- | --- | --- | --- | --- | --- | --- | --- | --- | --- |
|  | meteo | indices | mixed | meteo | indices | mixed | meteo | indices | mixed |
| FWI | ns | ** | ** | ns | ns | ns | ** | *** | *** |
| FFMC | ns | ** | * | ns | ns | ns | *** | *** | *** |
| DMC | ** | *** | *** | ns | ** | * | ** | ** | ** |
| DC | ** | ** | ** | * | ** | ** | * | *** | ** |
| KBDIsi | * | *** | *** | ns | * | * | * | ** | *** |
| Angstroem | ** | *** | *** | * | * | * | ** | *** | *** |
| FMI | * | *** | ** | ns | * | * | *** | *** | *** |
| Nesterov | ** | *** | *** | * | ** | ** | *** | *** | *** |

*** = p <0.001, ** = p <0.01, * = p <0.05, ns = not significant.
