# Supplementary material for: Modelling the Meteorological Forest Fire Niche in Heterogeneous Pyrologic Conditions
Source: PLoS One. 2015 Feb 13;10(2):e0116875. doi: 10.1371/journal.pone.0116875 (PMC4332634; doi:10.1371/journal.pone.0116875)
Supplement: S4 Table — The results are referred to the comparison between the best models based on the different variables combination using the logistic (a) and Maxent (b) approaches. (DOC) [file pone.0116875.s006.doc]

**Table S4.**

**Results of the Wilcoxon rank sum tests for the best models.**

The results are referred to the comparison between the best models based on the different variables combination using the logistic (a) and Maxent (b) approaches.

(a) logistic

|  | w | | | sa | | | sn | | |
| --- | --- | --- | --- | --- | --- | --- | --- | --- | --- |
|  | meteo | indices | mixed | meteo | indices | mixed | meteo | indices | mixed |
| meteo | - | ns | ** | - | ns | ns | - | ns | ns |
| indices | ns | - | ns | ns | - | ns | ns | - | ns |
| mixed | ** | ns | - | ns | ns | - | ns | ns | - |

(b) Maxent

|  | w | | | sa | | | sn | | |
| --- | --- | --- | --- | --- | --- | --- | --- | --- | --- |
|  | meteo | indices | mixed | meteo | indices | mixed | meteo | indices | mixed |
| meteo | - | ** | ** | - | ** | ** | - | ns | ns |
| indices | ** | - | ns | ** | - | ns | ns | - | * |
| mixed | ** | ns | - | ** | ns | - | ns | * | - |

** = p <0.01, * = p <0.05, ns = not significant.
